# Instrumenting gait assessment using the Kinect in people living with stroke: reliability and association with balance tests

**DOI:** 10.1186/s12984-015-0006-8

**Published:** 2015-02-12

**Authors:** Ross A Clark, Stephanie Vernon, Benjamin F Mentiplay, Kimberly J Miller, Jennifer L McGinley, Yong Hao Pua, Kade Paterson, Kelly J Bower

**Affiliations:** School of Exercise Science, Australian Catholic University, Melbourne, Australia; Department of Physical Therapy, University of British Columbia, Vancouver, Canada; School of Physiotherapy, University of Melbourne, Melbourne, Australia; Department of Physiotherapy, Singapore General Hospital, Singapore, Singapore; Department of Physiotherapy, Royal Melbourne Hospital, Melbourne, Australia

**Keywords:** Rehabilitation, Measurement, Brain injury, Walking, 10 meter walk test

## Abstract

**Background:**

The Microsoft Kinect has been used previously to assess spatiotemporal aspects of gait; however the reliability of this system for the assessment of people following stroke has not been established. This study examined the reliability and additional information that the Kinect provides when instrumenting a gait assessment in people living with stroke.

**Methods:**

The spatiotemporal variables of step length, step length asymmetry, foot swing velocity, foot swing velocity asymmetry, peak and mean gait speed and the percentage difference between the peak and mean gait speed were assessed during gait trials in 30 outpatients more than three months post-stroke and able to stand unsupported. Additional clinical assessments of functional reach (FR), step test (ST), 10 m walk test (10MWT) and the timed up and go (TUG) were performed, along with force platform instrumented assessments of center of pressure path length velocity during double-legged standing balance with eyes closed (DLEC), weight bearing asymmetry (WBA) and dynamic medial-lateral weight-shifting ability (MLWS). These tests were performed on two separate occasions, seven days apart for reliability assessment. Separate adjusted multiple regressions models for predicting scores on the clinical and force platform assessments were created using 1) the easily assessed clinically-derived gait variables 10MWT time and total number of steps; and 2) the Kinect-derived variables which were found to be reliable (ICC > 0.75) and not strongly correlated (Spearman’s ρ < 0.80) with each other (i.e. non-redundant).

**Results:**

Kinect-derived variables were found to be highly reliable (all ICCs > 0.80), but many were redundant. The final regression model using Kinect-derived variables consisted of the asymmetry scores, mean gait velocity, affected limb foot swing velocity and the difference between peak and mean gait velocity. In comparison with the clinically-derived regression model, the Kinect-derived model accounted for >15% more variance on the MLWS, ST and FR tests and scored similarly on all other measures.

**Conclusions:**

In conclusion, instrumenting gait using the Kinect is reliable and provides insight into the dynamic balance capacity of people living with stroke. This system provides a minimally intrusive method of examining potentially important gait characteristics in people living with stroke.

## Introduction

The ability to ambulate independently in the community is one of the most important rehabilitation outcomes for people following stroke [1]. Gait impairments are common following stroke, resulting in restrictions in activities of daily living and in the ability to integrate back into the community after discharge from hospital [1,2]. It is therefore important to assess gait function in this patient cohort, as it is considered a proxy measure of the patient’s overall physical function status and capacity to return to their previous lifestyle.

Clinical assessments of gait are often limited to measuring the total time taken to walk a set distance, for example the 10 m walk test (10MWT). From this information, gait velocity (or speed) can be calculated which allows for comparison to normative standards and important thresholds such as the minimum velocity required to safely cross roads [3]. Additional information from these tests, such as the number of steps performed, are also sometimes recorded to gain greater insight into the person’s functional ability [4,5].

Whilst these clinical assessments of gait function are informative, they lack the precision and data richness of instrumented methods that provide the kinematic and spatiotemporal aspects of the gait cycle [5]. Instrumented systems include marker-based three dimensional (3D) motion analysis using multiple cameras, body-mounted inertial monitoring unit sensors, and instrumented walkways such as the GAITRite [6]. Results derived from these systems, such as inter-limb gait asymmetry [7-9], may provide important information about dynamic balance control and underlying impairments which are associated with long term outcomes but cannot be derived from standard clinical assessments. Furthermore, these measures may better elucidate the outcomes of gait training programs aimed at improving symmetry and weight shifting ability [10]. By more precisely and accurately quantifying movement it may be possible to observe subtle changes in physical function that standard clinical assessments are not sensitive to. However, further population-level research is needed to determine the additive benefits of instrumenting gait assessment in a clinical setting.

Although these systems offer some benefits, real world clinical feasibility is limited due to factors such as cost, portability, training and time requirements. Recent evidence indicates that the Microsoft Kinect can be used to assess some spatiotemporal aspects of gait [6,11], including validation against a camera-based 3D motion analysis system for step length, foot swing velocity and inter-limb gait asymmetry in healthy young adults [6]. Although the Kinect may not provide the precision of a multiple camera or body-mounted sensor system, the low price (<US$100 for the Kinect camera, plus the cost of a computer if both devices are needed), widespread availability, small size and marker-less data collection and analysis capabilities offers unique potential for providing a more clinically feasible method of instrumenting gait assessments.

There is a paucity of research examining the reliability and potential usefulness of the Kinect for assessing gait in clinical populations. One prior study used the Kinect for instrumenting the timed up and go (TUG) test in stroke survivors, reporting the data obtained were reliable and provided additional and unique information over and above the standard clinical outcome assessment information [12]. However, due to the 3 m restriction on walking inherent in the TUG test, there was only a small focus on the gait aspects of the TUG. No examination of inter-limb step length or velocity asymmetries was performed, which may provide important information related to a patient’s dynamic balance capabilities. Furthermore, the data reported are unlikely to reflect typical walking patterns due to the acceleration and deceleration occurring within this restricted range.

The aim of the present study was to determine if instrumenting a gait assessment using the Kinect provides reliable and potentially valuable information in comparison with the standard clinical 10MWT assessment. This was examined by determining 1) the inter-session reliability of key outcome variables, 2) the redundancy these variables have with the clinical assessments and each other, and 3) whether these variables provide unique and important information about physical function to complement and extend the standard clinical assessment-derived outcome measures. We hypothesized that 1) the Kinect-derived measures would be highly reliable, 2) many variables would be redundant, however a core group of Kinect-derived measures would give unique information regarding physical function, and 3) when implemented into a regression model to determine association with clinical and instrumented assessments of gait and balance, the addition of specific combinations of non-redundant variables would outperform the clinically-derived results.

## Methods

### Participants

Thirty participants living with stroke were consecutively recruited from the Community Therapy Service at The Royal Melbourne Hospital. Participants must have been diagnosed with a non-cerebellar ischemic or hemorrhagic stroke >3 months prior to recruitment, be attending physiotherapy for balance or mobility dysfunction, be able to stand unsupported for >30 seconds, and have a Mini Mental State Examination score ≥20. Exclusion criteria were severe apraxia, severe dysphasia or any other medical condition that may impact their balance ability (e.g. severe joint pain, progressive neurological disorders or visual impairment). This research was given ethical approval from the Royal Melbourne Hospital and University of Melbourne ethics committees, and all participants provided written informed consent prior to testing. Participants completed two testing sessions at the hospital one week apart, with each session consisting of 1) standard clinical assessments of gait and dynamic balance, 2) an instrumented gait assessment, and 3) an instrumented assessment of static and dynamic balance. The participants in this study are the same as those from a previously published article [12].

### Standard clinical assessments of gait and balance

In order of completion, the clinical assessments were the 10MWT, TUG, Step Test (ST) and Functional Reach (FR) test, and were undertaken in accordance with previously published protocols [13-17]. These outcome measures were chosen as they are commonly used within Australian rehabilitation settings and present a range of dynamic balance activities. All assessments were performed twice on each of the two days, with the best trial used for analysis, with the exception of the ST which was performed once as per the standard testing protocol. The same standardized procedure was followed during each testing session. Participants wore shoes and could use gait aids and/or ankle-foot orthoses for the gait assessment and TUG only. Participants wore their usual clothing; however, if needed, tape was used to make pants more closely fitted at the knees and ankles in order to increase the accuracy of joint center detection using the Kinect.

### Instrumented assessment of gait

A Microsoft Xbox360 Kinect camera was used during the additional gait assessment, which was performed after the 10MWT, to obtain spatiotemporal and kinematic information from the participant. The Kinect integrates information from video and depth-sensing cameras to create a 3D representation of the field of view [18]. An artificial intelligence algorithm provided freely by Microsoft is then used to automatically locate and track the joint centers and major anatomical landmarks of the body [19]. This enables the camera to provide information on the 3D movement of the participant in close to real-time.

The Kinect gait assessment could not be performed concurrently with the 10MWT, as the end location of that test was in a position which did not allow for placement of the Kinect camera or access to an external power supply without creating a trip hazard. Consequently the Kinect gait assessment walkway was setup in an alternative space in the rehabilitation center, with a walkway length of 6 m. The Kinect camera placement and field of view was setup at the end of this walkway, and calibrated using a custom written software program prior to each testing session using a protocol described previously [6,20].

The participant was instructed to start at the beginning of the walkway and walk towards the Kinect camera, stopping 0.5 m in front of it. For this study the acceptable field of view was restricted to a range from 1.5 to 3.5 m from the Kinect. This distance allowed for a minimum of one full gait cycle (i.e. a complete stride) per limb to be recorded per walking trial. The 3D skeleton position data for each ankle and shoulder center (ie. the position in the middle of the sternum) were recorded throughout the two trials, and expressed relative to the Kinect camera. These data were acquired at their native sampling frequency, which is irregular and fluctuates around 30 Hz. To overcome the sampling irregularity issues of the Kinect, spline interpolation was used to resample the Kinect data to 100 Hz. Data was loaded into a custom program and filtered using a Daubechies 4 undecimated wavelet 3.125 Hz lowpass filter. The gait event time points for toe-off and ground contact were identified using a supervised automated analysis algorithm described previously [6], which is based on the velocity of the movement of the ankle joint center.

The outcome measures analysed and reported in this study are based on those previously observed to be reliable in healthy young people [6]. Specifically, these variables included step length and foot swing velocity for the affected (deemed the limb contralateral to the side of the stroke) and unaffected limb. Measures of mean and peak gait velocity during the trial were derived from the anterior displacement of the shoulder center throughout the field of view of the Kinect. The difference between the peak and mean velocity was also calculated as a measure of the forward progression variability, and expressed as a percentage score. Additionally, given the level of inter-limb asymmetry often present during gait after stroke, asymmetry ratio scores were calculated for each measure which compared the affected and unaffected limb [9]. These asymmetry scores were converted to an absolute value and expressed as a percentage. All Kinect-derived outcome measures were averaged across the two trials.

### Instrumented assessment of static and dynamic balance

Force platform-derived measures of static (quiet stance) and dynamic (movement over a fixed base of support) balance were performed after the standard clinical tests. Static balance tests involved standing double-legged balance with eyes closed (DLEC), standing weight bearing asymmetry (WBA) and dynamic balance involved a medial-lateral weight shifting (MLWS) test. Each test was performed on one (DLEC) or two (WBA and MLWS) Nintendo Wii Balance Boards (NWBB) with custom software used to record data, and in the case of the MLWS test, provide visual feedback. These tests have demonstrated high test-retest reliability (ICC = 0.82 to 0.98) in people with stroke [21]. The DLEC test was performed on the NWBB with feet a fixed distance apart (17 cm between heels and toe-out angle of 14°) [22] and eyes focused on a target on the wall in front. Data was collected for three trials of 30 seconds duration and the median score was used for analysis. The outcome measure for this test was total center of pressure path velocity in cm/s, and this was derived using a technique described previously [23]. WBA was assessed by having the participant stand still for 30 seconds with a foot on each NWBB with heels 17 cm apart and toe-out angle of 14°. The difference in force distributed between the lower limbs expressed as a percentage of body mass was deemed the WBA. This data collection and analysis technique is similar to previously published articles examining asymmetry with multiple NWBBs [24,25]. The MLWS test was designed to measure the ability to repeatedly shift body weight distribution between the lower limbs to follow a visual feedback target for 30 seconds. Participants were required to shift their body weight alternatively between the left and right sides to reach and hold (for one second) in a target area (equating to 60-80% body weight) displayed as columns on a television screen. This test was based on a previous design shown to be responsive to change post-stroke [26] and has been used as an outcome measure in a recent stroke rehabilitation trial [27]. Data (number of shifts completed in 30 seconds) were collected for five trials and the median of the last three trials was used [17].

### Statistical analysis

Intraclass correlation coefficients (ICC_2,k_) were used as indices of relative reliability of the Kinect-derived variables, and these coefficients were calculated in a 2-way analysis of variance (ANOVA) based on absolute agreement. Absolute reliability was represented by the standard error of measurement (SEM), which was derived from the square root of the mean square error term from the respective repeated ANOVA, and the minimum detectable change (MDC_95_) score.

To compare the abilities of the standard clinical assessment of gait versus a Kinect instrumented gait assessment for predicting static and dynamic balance scores, we created two independent regression models using data from the first testing session. The first model only included the clinically-derived gait assessment outcome measures, and included two independent variables: 1) the time taken to complete the 10MWT, and 2) the number of steps taken during the test. The second model consisted of the Kinect-derived outcome variables. To minimize model overfitting and to avoid multicollinearity from correlated variables, we *a priori* specified that Kinect-derived variables which were strongly related (Spearman *ρ*’s ≥ 0.80) to either of the clinical gait assessment outcome variables would be deemed redundant and excluded from the model. The exception was mean gait velocity recorded during the trial, which was retained as the proxy Kinect-derived measure for 10MWT time. We compared the two (non-nested) models using the model unadjusted and adjusted R^2^ values [28], and additionally the Akaike Information Criterion (AIC) [29] which is a penalized measure of model fit.

## Results

All 30 participants attended both testing sessions. Table 1 provides the participant demographics. Ten of the thirty participants required the use of gait aids during the 10MWT. Of these ten, two could not perform the test without using a 4-wheeled walking frame. The data for these two subjects was retained for assessing the reliability of gait velocity derived from the Kinect, but were excluded from the regression and redundancy analysis because accurate information regarding all Kinect-derived variables except those calculated from the shoulder center could not be obtained. The reliability results for the Kinect-derived gait measures are provided in Table 2. All variables were found to have high reliability (all ICC scores ≥ 0.80).

**Table 1:** **Participants’ characteristics recorded during the first assessment session**

**Demographic Variable**	**Result**
Age (yrs)	68 ± 15
Height (cm)	166.7 ± 9.4
Body Mass (kg)	72.5 ± 11.9
Gender (n = male)	21
Mini-mental State exam score (/30)	27.1 ± 2.7
Lesion type (% Infarct)	73
Lesion side (% Right)	63
Time since stroke (months)	21 ± 19
Hypertension (%)	70
Dyslipidaemia (%)	47
Type 2 Diabetes mellitus (%)	27
10 m Walk test speed (m/s)	0.97 ± 0.35
10 m Walk test steps (n)	20 ± 9
Timed up and go (s)	17.7 ± 10.5
Functional reach (cm)	28.5 ± 7.4
Step test (n)	9.3 ± 4.9
Static double leg eyes closed balance – Path velocity (cm/s)	2.06 ± 1.05
Static weight-bearing asymmetry (% Body mass)	46.3 ± 8.5
Medial-lateral weight shifting ability (no. of shifts in 30 seconds)	10.0 ± 3.7

Values are reported as mean ± standard deviation unless otherwise indicated.

**Table 2:** **Test-retest reliability measures for the Kinect-derived gait variables**

**Variables**	**Test**	**Retest**	**ICC(2,**_**k**_ **)**	**SEM**	**MDC**_**95**_
	**Mean ± SD**	**Mean ± SD**	**(95% CI)**		
Affected step length (mm)	507 ± 147	513 ± 169	0.97 (0.95 to 0.99)	29	80
Unaffected step length (mm)	520 ± 166	520 ± 144	0.98 (0.96 to 0.99)	21	58
Step length asymmetry (%)	15.0 ± 15.6	17.4 ± 19.4	0.89 (0.76 to 0.95)	5.2	14.3
Affected foot swing velocity (m/s)	3.18 ± 0.91	3.16 ± 0.90	0.97 (0.94 to 0.99)	0.16	0.44
Unaffected foot swing velocity (m/s)	3.08 ± 1.3	3.09 ± 1.29	0.99 (0.97 to 0.99)	0.13	0.36
Foot swing velocity asymmetry (%)	12.6 ± 13.7	14.0 ± 13.0	0.80 (0.58 to 0.90)	6.1	17.0
Mean velocity (m/s)	0.89 ± 0.33	0.90 ± 0.32	0.98 (0.96 to 0.99)	0.05	0.13
Peak velocity (m/s)	1.22 ± 0.39	1.22 ± 0.39	0.98 (0.95 to 0.99)	0.06	0.15
Peak – Mean velocity difference (%)	41.8 ± 18.4	39.8 ± 18.1	0.96 (0.92 to 0.98)	3.7	10.2

Abbreviations: SD = standard deviation; ICC = intraclass correlation coefficient; CI = confidence interval; SEM = standard error of measurement; MDC = minimum detectable change.

Redundancy analysis of the Kinect-derived gait variables with the clinical measures of 10MWT time and number of steps revealed that four of the nine variables were highly correlated (i.e. redundant), and therefore these measures were excluded from any further analysis (Table 3). Redundancy analysis of the remaining Kinect-derived outcome measures with each other is provided in Table 4. This revealed that foot swing velocity for the affected and unaffected limbs was highly correlated. A decision was made to discard the unaffected limb foot swing velocity from any further analysis, as the affected limb score may be a more likely target for interventions such as botulinum toxin injection [30]. Consequently, only four variables remained to be inputted into the regression model, namely 1) step length asymmetry, 2) foot swing velocity asymmetry, 3) the difference between peak and mean gait velocity, and 4) affected limb foot swing velocity. A fifth input, mean gait velocity, was included into the model to represent the standard clinical measure of gait speed.

**Table 3:** **Redundancy analysis of the Kinect-derived gait variables compared to the clinical measures of manually assessed time (MT) and manually assessed number of steps (MS) using Spearman’s correlation**

**Outcome Measure**	**MT**	**MS**
Affected step length (mm)	−0.86*	−0.93*
Unaffected step length (mm)	−0.93*	−0.96*
Step length asymmetry (%)	0.45*	0.39
Affected foot swing velocity (mm/s)	−0.71*	−0.76*
Unaffected foot swing velocity (mm/s)	−0.78*	−0.80*
Foot swing velocity asymmetry (%)	0.25	0.20
Mean velocity (mm/s)	−0.92*	−0.92*
Peak velocity (mm/s)	−0.92*	−0.93*
Peak – Mean velocity difference (%)	0.66*	0.65*

*significant at P < 0.05.

The correlation between MT and MS is 0.93*.

**Table 4:** **Redundancy analysis of the Kinect-derived gait variables using Spearman’s correlation matrix**

**Variables**	**Step length**	**Affected foot**	**Unaffected foot swing velocity**	**Foot swing velocity asymmetry**
	**Asymmetry**	**Swing velocity**		
Peak – Mean velocity difference	0.53*	−0.59*	−0.60*	0.21
Foot swing velocity asymmetry	0.02	0.06	−0.16	
Unaffected foot swing velocity	−0.26	0.91*		
Affected foot swing velocity	−0.31			

*significant at P < 0.05.

Table 5 describes the results for the regression models. Both the clinically-derived and Kinect-derived models produced similar adjusted R^2^ values for the TUG, DLEC and WBA tests (R^2^ value differences of 0.06, 0.01 and 0.06 respectively). In contrast, the Kinect-derived model outperformed the clinically-derived model for predicting scores on the MLWS, ST and FR by R^2^ value differences of 0.25, 0.21 and 0.19 respectively. Furthermore, the AIC values were lower in four of the six Kinect-derived models, indicating that the Kinect-derived models had better overall model fit.

**Table 5:** **Results for the clinically-derived and Kinect-derived multiple regression models**

**Functional assessment**	**Clinical**			**Kinect**		
	**R** ^**2**^	**Adj R** ^**2**^	**AIC**	**R** ^**2**^	**Adj R** ^**2**^	**AIC**
*Overall physical function*						
Timed up and go	0.89	0.88**	81	0.95	0.94**	63
*Static balance*						
Static double leg eyes closed balance	0.41	0.37*	−8	0.47	0.36*	−5
Static weight-bearing asymmetry	0.49	0.44**	102	0.50	0.38*	108
*Dynamic balance*						
Functional reach	0.43	0.39*	109	0.65	0.58**	100
Step test	0.61	0.58**	70	0.83	0.79**	52
Medial-lateral weight shifting ability	0.48	0.44**	60	0.75	0.69**	46

Clinically-derived model included 2 input variables: 10MWT time; total number of steps. Kinect-derived model included 5 input variables: Mean velocity; Foot swing velocity asymmetry; Peak – Mean velocity difference; Step length asymmetry; Affected foot swing velocity.

*p-values <0.01; **p-values <0.001.

R^2^, Ezekiel equation adjusted R^2^ and the Akaike Information Criterion (AIC) score are provided for comparison of model strengths.

## Discussion

The present study examined the instrumentation of a gait assessment in a clinical stroke rehabilitation setting using the Microsoft Kinect. All outcome measures derived from the Kinect were found to be highly reliable (ICC ≥ 0.80) in accordance with hypothesis 1. Supporting hypothesis 2, many of the outcome measures were redundant within device or when compared to the 10MWT time and the total number of steps performed. Interestingly, when a combination of non-redundant Kinect-derived variables were inputted into a multiple regression model to predict dynamic balance assessments, this model had higher adjusted R^2^ values than one built using the 10MWT time taken to complete and the total number of steps performed. Specifically, the Kinect-derived model explained >15% more variance for the FR (Kinect-derived adjusted R^2^ = 0.58, clinically-derived adjusted R^2^ = 0.39; 19% more variance explained), ST (Kinect-derived adjusted R^2^ = 0.79, clinically-derived adjusted R^2^ = 0.58; 21% more variance explained) and MLWS assessments (Kinect-derived adjusted R^2^ = 0.70, clinically-derived adjusted R^2^ = 0.44; 26% more variance explained). Conversely, the model results were similar (≤6% more variance explained by either model) for the static balance tests DLEC (Kinect-derived adjusted R^2^ = 0.36, clinically-derived adjusted R^2^ = 0.37; 1% less variance explained), WBA (Kinect-derived adjusted R^2^ = 0.38, clinically-derived adjusted R^2^ = 0.44; 6% less variance explained), and the overall measure of physical function test the TUG (Kinect-derived adjusted R^2^ = 0.94, clinically-derived adjusted R^2^ = 0.88; 6% more variance explained). This provides partial support for hypothesis 3, but only for the dynamic balance assessments.

In regard to reliability, all outcome measures recorded acceptable ICC scores, and seven of the nine variables possessed ICC values > 0.90. This was expected, as the variables were chosen based on those reported previously to be reliable using the Kinect to assess gait, as well as other systems assessing spatiotemporal gait parameters in people living with stroke [6,8,31]. However, in the present study the participants were not required to perform the assessments barefoot and while wearing shorts. Consequently, this is the first study to show that the Kinect can provide reliable spatiotemporal gait information in a stroke population assessed in a clinical setting with minimal interference to the patient. This is important, because instrumenting gait assessments using the Kinect allows for examination of inter-limb step length and foot swing velocity asymmetries – measures which cannot currently be readily assessed in a clinical setting. Additionally, while pressure-mat systems such as the GAITRite can provide some spatiotemporal measures of gait such as stride length, unlike the Kinect they cannot be used to examine other potentially important factors such as foot swing velocity or variability in trunk motion.

Instrumenting a gait assessment using the Kinect allowed for improved prediction of scores on the FR, ST and MLWS tests compared to the non-instrumented gait assessment. When using the often reported R value thresholds of poor (R < 0.40), modest (R = 0.40 – 0.74) or excellent (R ≥ 0.75) [32], the clinical assessment model was rated as fair for the FR and MLWS tests and narrowly exceeded the threshold for excellent for the ST. In contrast, the Kinect-derived model was rated as excellent for all three of these tests of dynamic balance capability during controlled movements. The large difference in predictive strength between the two regression models indicates that the standard clinical assessment of gait is not suitable as a strong proxy measure of dynamic balance. This supports prior research showing only moderate to low correlations between these two aspects of physical function in people with Parkinson’s disease or the community-dwelling elderly [33,34]. Instrumenting a gait assessment using the Kinect may allow for large scale screening which provides insight into balance whilst also obtaining information on gait from a single test. However, the utility of this assessment would be highly context dependent and not suitable as a replacement for a thorough assessment of balance where indicated. Of particular interest from the present study is the disparity in the strength of the association that the two different models have with MLWS performance. Previous studies have highlighted the potential for this assessment to provide insight into dynamic balance [26,27]; however, it requires two force platforms and a computer monitor to provide visual feedback, which reduces its clinical feasibility. Having a single Kinect setup to acquire data during a gait trial without the need to provide visual feedback is much simpler to implement. Whilst the Kinect-derived model was capable of predicting performance on the dynamic balance assessments, neither regression model was able to accurately predict performance on the static balance assessments of WBA and standing still with eyes closed. This provides further evidence that static balance capability has only a limited association with gait function, and supports the work by Lewek et al. [35] which found that spatiotemporal gait parameters are more closely related to dynamic rather than static balance in people with chronic stroke.

This study builds on our previous work which instrumented the TUG using the Kinect in a stroke population [12]. Similar to this present study, the prior work examined individual components of the TUG and observed that this provided additional information over-and-above that which can be obtained from just recording the total time taken to complete the test. The present study differs in that it focuses on the spatiotemporal components of steady-state gait, which cannot be derived from the short walking distance of the TUG. Given our small sample size, we were unable to statistically contrast the predictive validity of the Kinect-TUG and Kinect-Gait variables. However, we noted that both sets of Kinect variables (i) were non-redundant with their corresponding clinical measures, (ii) provided incremental predictive values over their corresponding clinical measures, and (iii) produced similar model R^2^ values (ST R^2^: Kinect-TUG = 0.73, Kinect-gait = 0.79; FR R^2^: Kinect-TUG = 0.53, Kinect-gait = 0.58). The major advantages of instrumenting a gait assessment in comparison with the TUG are that it can be done with no additional equipment (i.e. the standardised height chair of the TUG), increasing its potential utility, and by any patient who can walk a relatively short distance, making it potentially more generalizable to an in patient severe stroke population. The present study also builds on the prior study by examining the association of the Kinect-derived variables with both instrumented and clinical assessments of static and dynamic balance, with the finding that spatiotemporal aspects of gait are more strongly associated with dynamic rather than static balance.

This study had limitations. The study sample consisted of a relatively small and heterogenous group of stroke survivors recruited from a single outpatient facility. The allowance of gait aid use and inclusion of participants with minimal gait deficits may have impacted on the strength of associations found. Outcome measures were limited to spatiotemporal variables which have been previously identified as being reliable. Potentially important outcome measures such as medial-lateral center of mass sway [36] were not examined, and if reliable their inclusion may have strengthened the regression models. These variables were not analyzed for a number of reasons related to potential data error. For example, medial-lateral center of mass sway is highly reliant on accurate identification of the hip center by the skeleton tracking algorithm, however in this patient population this landmark was often occluded due to reasons such as elbow flexion of the stroke-affected arm or handles on gait assistive devices. Furthermore, additional Kinect-based measures were not analyzed because of the already high number of variables derived from the Kinect for this form of regression research. Another limitation was the accuracy and precision of the Kinect, which is unlikely to be as high as body-mounted sensors or marker-based 3D motion analysis camera systems. However, given the potential clinical feasibility and mass physical function screening advantages that this system offers, we believe that its possible benefits warrant further investigation. The use of the manually assessed time and manually assessed number of steps together in the clinically-derived regression model is also a limitation, as correlation analysis revealed these two variables to be somewhat redundant (Spearman’s ρ = 0.93). This may have unfairly penalised the results of the clinically-derived model given the two somewhat similar input variables, biasing the study towards a positive outcome for the Kinect assessment. However, performing the regression analysis using just manually assessed time to complete the test as the sole input variable did not create a meaningful change in the adjusted R^2^ values (R^2^ value change range = −0.02 to 0.02), and therefore we are confident in our observations. The restricted field of view of the Kinect does not allow for multiple gait cycles to be examined from a single test. This is a limitation when it comes to assessing asymmetry, or measures of gait variability. Also potentially related to this restricted field of view, we observed a difference in the gait speed derived from the Kinect compared to the stopwatch, with the Kinect-derived value being slower (median [inter-quartile range] difference between the 10MWT and Kinect gait speeds for each individual = 7.8 [1.5 to 17.5] %). Visual examination of our data revealed that for some participants deceleration occurred as they approached the camera, but in the majority the mean gait speed was relatively stable. Consequently, in the former case this disparity may have been a result of patients beginning gait deceleration while they were still in the field of view. In the latter example however, the participant may have simply been walking slower with a camera located at the end of the test in contrast to the 10MWT which finished multiple meters from any obstruction. Both of these strategies may impact gait velocity and step length data, and therefore should be considered when comparing results obtained from the Kinect with those of other methods. Finally, our patient cohort was reasonably high functioning, with an average gait speed well above that considered critical for community ambulation. Whether the Kinect provides useful information in people with slower walking speeds (i.e. poorer function) cannot be generalized from our findings. This is an important question as these people may be at most risk of adverse outcomes related to physical function.

In conclusion, these findings provide support for the potential usefulness of implementing a Kinect instrumented gait assessment in a clinical setting. This single, easy to implement and reliable assessment may allow for greater insights into a person's gait and dynamic balance ability. Variables derived from this assessment may allow for better monitoring of change in gait performance over time in both clinical and research settings, as well as providing information to inform targeted treatment approaches. It is important that future research examines ways to optimize the feasibility and utility of this system in a clinical setting to ensure translation into standard clinical practice.

## References

[1] van de Port IG, Kwakkel G, Lindeman E (2008). Community ambulation in patients with chronic stroke: how is it related to gait speed?. J Rehabil Med.

[2] Salbach NM, Mayo NE, Higgins J, Ahmed S, Finch LE, Richards CL (2001). Responsiveness and predictability of gait speed and other disability measures in acute stroke. Arch Phys Med Rehabil.

[3] Asher L, Aresu M, Falaschetti E, Mindell J (2012). Most older pedestrians are unable to cross the road in time: a cross-sectional study. Age Ageing.

[4] Sturt R, Holland A, New P (2009). Walking ability at discharge from inpatient rehabilitation in a cohort of non-traumatic spinal cord injury patients. Spinal Cord.

[5] Coutts F (1999). Gait analysis in the therapeutic environment. Man Therap.

[6] Clark RA, Bower KJ, Mentiplay BF, Paterson K, Pua YH (2013). Concurrent validity of the Microsoft Kinect for assessment of spatiotemporal gait variables. J Biomech.

[7] Hendrickson J, Patterson KK, Inness EL, McIlroy WE, Mansfield A (2014). Relationship between asymmetry of quiet standing balance control and walking post-stroke. Gait Posture.

[8] Lewek MD, Randall EP (2011). Reliability of spatiotemporal asymmetry during overground walking for individuals following chronic stroke. J Neurologic Physical Ther.

[9] Patterson KK, Gage WH, Brooks D, Black SE, McIlroy WE (2010). Evaluation of gait symmetry after stroke: A comparison of current methods and recommendations for standardization. Gait Posture.

[10] Patterson KK, Mansfield A, Biasin L, Brunton K, Inness EL, McIlroy WE (2014). Longitudinal Changes in Poststroke Spatiotemporal Gait Asymmetry Over Inpatient Rehabilitation. Neurorehab Neural Repair.

[11] Stone E, Skubic M (2011). Evaluation of an inexpensive depth camera for in-home gait assessment. J Ambient Intell Smart Environ.

[12] Vernon S, Paterson K, Bower K, McGinley J, Miller K, Pua Y (2014). Quantifying Individual Components of the timed up and go using the Kinect in people living with stroke. Neurorehab Neural Repair.

[13] Bernhardt J, Ellis P, Denisenko S, Hill K (1998). Changes in balance and locomotion measures during rehabilitation following stroke. Physiother Res Int.

[14] Thrane G, Joakimsen RM, Thornquist E (2007). The association between timed up and go test and history of falls: the Tromso study. BMC Geriatr.

[15] Mercer VS, Freburger JK, Chang SH, Purser JL (2009). Step test scores are related to measures of activity and participation in the first 6 months after stroke. Phys Ther.

[16] Duncan P, Weiner D, Chandler J, Studenski S (1990). Functional reach: a new clinical measure of balance. J Gerontol.

[17] Bower K, Clark RA, Martin C, Miller K. Clinical feasibility of the Nintendo Wii for balance training post-stroke: A phase II randomised controlled trial in an inpatient setting. Clin Rehabil. 2014;28:912–923. 10.1177/0269215514527597 24668359

[18] Menna F, Remondino F, Battisti R, Nocerino E (2011). Geometric Investigation of a Gaming Active Device.

[19] Shotton J, Fitzgibbon A, Cook M, Sharp T, Finocchio M, Moore R (2011). Real-Time Human Pose Recognition in Parts from Single Depth Images. Computer Vision and Pattern Recognition (CVPR), 2011 IEEE Conference; 20–25 June 2011.

[20] Clark RA, Pua Y-H, Fortin K, Ritchie C, Webster KE, Denehy L (2012). Validity of the Microsoft Kinect for assessment of postural control. Gait Posture.

[21] Bower K, McGinely J, Miller K, Clark RA (2014). Instrumented static and dynamic balance assessment after stroke using Wii Balance Boards: Reliability and association with clinical tests. PLOS One.

[22] McIlroy WE, Maki BE (1997). Preferred placement of the feet during quiet stance: Development of a standardized foot placement for balance testing. Clin Biomech.

[23] Clark RA, Bryant AL, Pua Y, McCrory P, Bennell K, Hunt M (2010). Validity and reliability of the Nintendo Wii Balance Board for assessment of standing balance. Gait Posture.

[24] Clark RA, McGough R, Paterson K (2011). Reliability of an inexpensive and portable dynamic weight bearing asymmetry assessment system incorporating dual Nintendo Wii Balance Boards. Gait Posture.

[25] Foo J, Paterson K, Williams G, Clark R (2013). Low-cost evaluation and real-time feedback of static and dynamic weight bearing asymmetry in patients undergoing in-patient physiotherapy rehabilitation for neurological conditions. J NeuroEng Rehab.

[26] de Haart M, Geurts AC, Dault MC, Nienhuis B, Duysens J (2005). Restoration of weight-shifting capacity in patients with postacute stroke: a rehabilitation cohort study. Arch Phys Med Rehabil.

[27] Bower K, Clark RA, McGinley J, Martin C, Miller K (2013). Feasibility and efficacy of the Nintendo Wii™ gaming system to improve balance performance post-stroke: Protocol of a phase II randomised controlled trial in an inpatient rehabilitation setting. Games Health.

[28] Ezekiel M. Methods of Correlation Analysis. New York: Wiley; London, Chapman. 1930.

[29] Akaike H (1974). A new look at the statistical model identification. Automatic Control, IEEE Transactions on.

[30] Gracies JM, Elovic E, McGuire J, Simpson D (1997). Traditional pharmacological treatments for spasticity part I: Local treatments. Muscle Nerve.

[31] Høyer E, Opheim A, Strand LI, Moe-Nilssen R (2014). Temporal and spatial gait parameters in patients dependent on walking assistance after stroke: Reliability and agreement between simple and advanced methods of assessment. Gait Posture.

[32] Fleiss J (1986). The Design and Analysis of Clinical Experiments.

[33] Desai A, Goodman V, Kapadia N, Shay BL, Szturm T (2010). Relationship between dynamic balance measures and functional performance in community-dwelling elderly people. Phys Ther.

[34] Yang YR, Lee YY, Cheng SJ, Lin PY, Wang RY (2008). Relationships between gait and dynamic balance in early Parkinson's disease. Gait Posture.

[35] Lewek MD, Bradley CE, Wutzke CJ, Zinder SM (2014). The relationship between spatiotemporal gait asymmetry and balance in individuals with chronic stroke. J Appl Biomech.

[36] Hodt-Billington C, Helbostad JL, Moe-Nilssen R (2008). Should trunk movement or footfall parameters quantify gait asymmetry in chronic stroke patients?. Gait Posture.

